# *Streptomyces albidoflavus* Strain CARA17 as a Biocontrol Agent against Fungal Soil-Borne Pathogens of Fennel Plants

**DOI:** 10.3390/plants11111420

**Published:** 2022-05-26

**Authors:** Antonia Carlucci, Maria Luisa Raimondo, Donato Colucci, Francesco Lops

**Affiliations:** Department of Agricultural Sciences, Food, Natural Resources and Engineering, University of Foggia, Via Napoli 25, 71122 Foggia, Italy; donatocolucci.dc@gmail.com (D.C.); francesco.lops@unifg.it (F.L.)

**Keywords:** microbial antagonist, *Athelia rolfsii*, *Fusarium oxysporum*, *Plectosphaerella ramiseptata*, *Sclerotinia sclerotiorum*, *Verticillium dahliae*, biological control

## Abstract

Fennel crop is a horticultural plant susceptible to several soil-borne fungal pathogens responsible for yield losses. The control of fungal diseases occurring on fennel crops is very difficult with conventional and/or integrated means; although several chemical fungicides are able to contain specific fungal diseases, they are not registered for fennel crops. The intensive use of some fungicides causes public concern over the environment and human health. The main aims of this study were to assess the ability of a strain of *Streptomyces albidoflavus* CARA17 to inhibit the growth of fungal soil-borne pathogens, and to protect fennel plants against severe fungal soil-borne pathogens such as *Athelia rolfsii*, *Fusarium oxysporum*, *Plectosphaerella ramiseptata*, *Sclerotinia sclerotiorum* and *Verticillium dahliae*. This study confirmed that the CARA17 strain has been able to inhibit the mycelium growth of pathogens in vitro conditions with significant inhibition degrees, where *S. sclerotiorum* resulted in being the most controlled. The strain CARA17 was also able to significantly protect the fennel seedlings against fungal soil-borne pathogens used in vivo conditions, where the treatment with an antagonist strain by dipping resulted in being more effective at limiting the disease severity of each fungal soil-borne pathogen. Moreover, any treatment with the CARA17 strain, carried out by dipping or after transplanting, produced benefits for the biomass of fennel seedlings, showing significant effects as a promoter of plant growth. Finally, the results obtained showed that CARA17 is a valid strain as a biocontrol agent (BCA) against relevant fungal soil-borne pathogens, although further studies are recommended to confirm these preliminary results. Finally, this study allowed for first time worldwide the association of *Plectosphaerella ramiseptata* with fennel plants as a severe pathogen.

## 1. Introduction

Fennel (*Phoeniculum vulgare* Mill) is a native horticultural plant of the Southern Europe and Mediterranean area. In particular, in Italy, this crop is of great economic importance, with about 85% of the world production consisting of 507,054 tons [1]. Many soil-borne fungi, reported throughout world, are severe pathogens for fennel crops, causing general symptoms of decline such as root and stem rot (*Rhizoctonia solani*, *Sclerotinia sclerotiorum*, *Athelia rolfsii*) [2,3,4], vascular diseases (*Verticillium dahliae*, *Fusarium oxysporum*) [2,5] and damping off of seedlings (*Pythium aphanidermatum*) [4]. These fungal pathogens are all notoriously difficult to control with conventional and/or integrated management approaches. To date, the advice for a good management is based on the use of resistant varieties, chemical treatment of seeds, rotation of crops, and the solar treatment of nursery beds to reduce the inoculums of pathogens in the soil. Several fungicides have been reported as being effective against the mentioned soil-borne fungi, but none is specifically registered to control or prevent the disease and/or symptoms in fennel crops. Moreover, intensive use of fungicides in agriculture has raised public concern over the environment and human health [6].

For these reasons, several researchers have focused on biological control as a promising alternative approach to controlling soil-borne diseases in sustainable and organic agriculture. Indeed, many microbial antagonists have been demonstrated to be able to control a large number of pathogens such as fungi and bacteria. For instance, *Trichoderma* spp. as biocontrol agents (BCA) are the most important and are more used against different fungal pathogens such as *Sclerotinia sclerotiorum* and *Phytophthora nicotianae* [7] and *Chalara thielavioides* [8]; *Pseudomonas* spp. is used against *Verticillium dahliae* [9] and *Bacillus* spp. against several phytopathogens [10]. Moreover, recently, plant growth promoting rhizobacteria (PGPR) have been demonstrated to promote plant growth, support nutrition and suppress different plant diseases [11], especially those that are gram-positive and endospore-forming due to being resistant to heat, drying, radiation and toxic chemicals [12,13]. In particular, *Streptomyces* spp. are the most spread gram-positive filamentous bacteria that are ubiquitous in the soil as free-living organisms and symbionts of plants and animals [14]. They are known for producing a wide variety of active biological compounds and are used in agriculture as plant growth promoters, and to be effective as a BCA against a large number of plant pathogens [15,16].

Although a few studies have been carried out to assess the biocontrol efficacy and mechanism of *Streptomyces* spp. [17,18], to date there has been no research related to the control of soil-borne pathogens on fennel crops.

Therefore, the main aims of the present study were: (i) to identify the *Streptomyces* strain isolated and used in this study using molecular tools; (ii) to assess its antagonistic and biostimulant activities; (iii) to assess its putative toxicity/pathogenicity on cucumber cotyledons; (iv) to determine its biocontrol efficacy in vitro and in vivo conditions on fennel seedlings against five fungal soil-borne pathogens, *Athelia rolfsii*, *Fusarium oxysporum*, *Plectosphaerella ramiseptata*, *Sclerotinia sclerotiorum* and *Verticillium dahliae*.

## 2. Results

### 2.1. Morphological and Molecular Identification

The amplicon of the CARA17 strain obtained during PCR analysis produced a fragment of 669 bp. The sequence of this amplicon analyzed in GenBank (Acc. n. ON548415) resulted in having a similarity of 100% with *Streptomyces albidoflavus* group.

The ITS sequence of the *Plectosphaerella* strain analyzed in GenBank by the BLAST tool resulted in having a similarity of 100% with the reference strain of *Plectosphaerella ramiseptata* (CBS 131861; Acc. n. JQ246953).

### 2.2. Inhibitory Activity against Fungal Pathogens in Dual Cultures

According to Shapiro–Wilk tests, the data followed normal distributions for all CARA17 exposition times (14, 21 and 28 days), with W values of 0.77, 0.79 and 0.78 (*p* < 0.05), respectively. The Levene tests showed that the homogeneity of variance was also significant for all three CARA17 exposition times (F = 3.65/3.64/4.19; *p* < 0.05).

**Figure 1:**
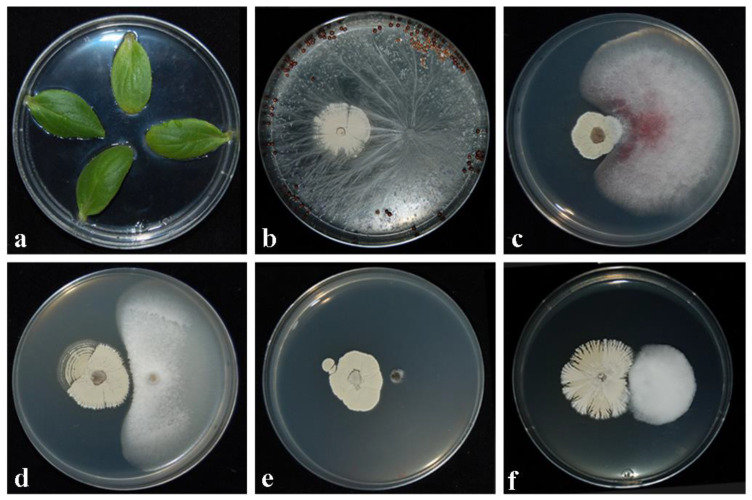
Phytotoxicity/pathogenicity assays carried out on young leaf cotyledones of cucumber (**a**), and inhibition activity played by CARA17 strain against *Athelia rolfsii* (**b**), *Fusarium oxysporum* (**c**), *Plectosphaerella ramiseptata* (**d**), *Sclerotinia sclerotiorum* (**e**), and *Verticillium dahliae* (**f**) after 28 days of exposition of fungal pathogens vs. CARA17 strain grown on medium.

One-way ANOVA demonstrated that significant differences in inhibitory activity (IA) by the CARA17 strain against fungal pathogens were observed. The IAs played by the CARA17 strain in dual cultures with fungal pathogens is reported in Table 1. It is possible to observe that the percentage values of IA decreased from 14 at 28 days after inoculation with CARA17 on a medium. In general, the *Streptomyces* strain was able to inhibit the mycelial growth of all fungal pathogens tested with variable percentage values of IA. *Sclerotina sclerotiorum* was the most sensible to antagonistic activity played by the CARA17 strain, as the IAs recorded were 100% at 14, 21 and 28 days after its inoculation. *Athelia rolfsii* resulted in being the least inhibited by the CARA17 strain at all inoculation times. The remaining fungal soil-borne pathogens, *V. dahliae*, *F. oxysporum* and *P. ramiseptata*, resulted in being well controlled with percentage values of IA, such as 71.30% (*V. dahliae*), 82.73% (*P. ramiseptata*) and 84.71% (*F. oxysporum*) after 28 days of inoculation (Table 1; Figure 1). 

### 2.3. Assessment of Toxicity/Pathogenicity of CARA17 on Cucumis sativus L. cotyledons In Vitro

To ascertain if the CARA17 strain was phytotoxic for fennel seedlings, no symptoms were observed after 3, 6 and 12 days from inoculations when aliquots of its propagules were placed on young cotyledons of cucumber (Figure 1).

### 2.4. Assessment of Antagonistic Effectiveness of CARA17 Strain on Foeniculum vulgare L. Seedlings against Fungal Soil-Borne Pathogens In Vivo

**Figure 2:**
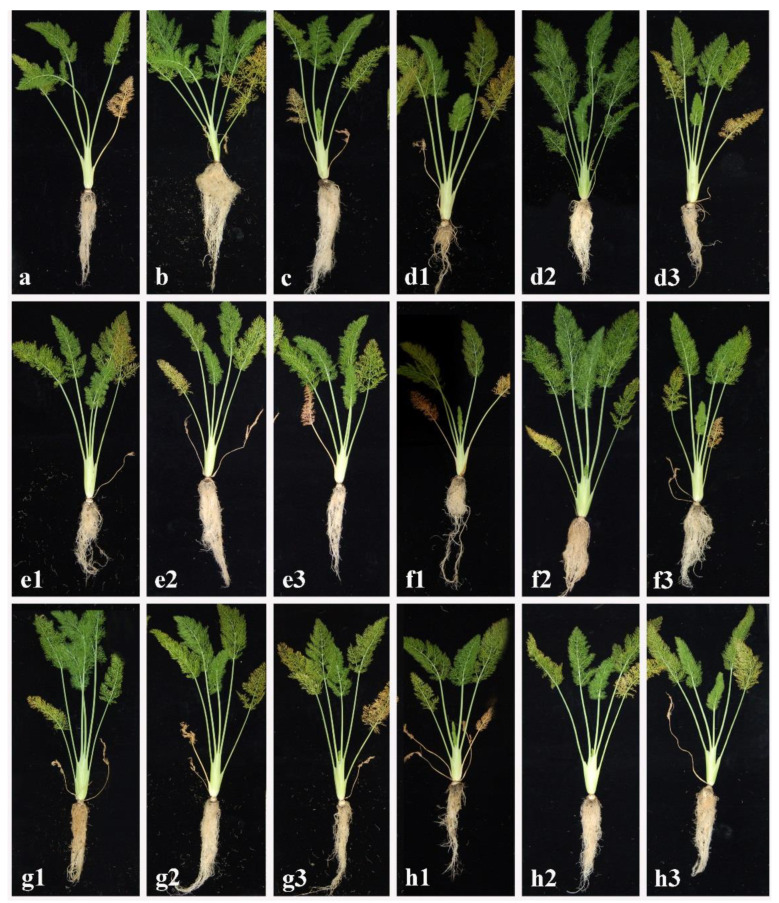
Effectiveness of CARA17 strain as antagonist for controlling disease severity by dipping treatment and after transplanting of fennel seedlings. Fennel seedlings not treated (**a**), treated with CARA17 strain by dipping (**b**) and after transplanting (**c**). Fennel seedlings treated by only fungal pathogen (1); fungal pathogen with CARA17 strain by dipping (2) and after transplanting (3). Inoculation with: *Athelia rolfsii* (**d1**–**d3**); *Fusarium oxysporum* (**e1**–**e3**); *Plectosphaerella ramiseptata* (**f1**–**f3**); *Sclerotinia sclerotiorum* (**g1**–**g3**); *Verticillium dahliae* (**h1**–**h3**).

The results obtained from in vivo experiments carried out on fennel seedlings are reported in Table 2. The symptoms observed on the root consisted of browning, rot, and growth reduction, while on the leaves they consisted of general growth reduction and yellowing of the epigeal portion (Figure 2). The DS values collected by observations carried out on the root of seedlings treated by dipping in CARA17 inoculum suspension were 0.0 for *A. rolfsii* and *S. sclerotiorum*, from 0.6 to 0.9 for *P. ramiseptata*, *V. dahliae* and *F. oxysporum*. Disease severity values observed on roots increased for experiments where the CARA17 strain was added to a pot after the transplanting of fennel seedlings. In particular, DS values varied from 0.6 to 1.9 against *S. sclerotiorum*, *V. dahliae*, *F. oxysporum* and *P. ramiseptata*. No DS value was produced by *A. rolfsii.* The DS values were higher when no CARA17 inoculum was used against the fungal soil-borne pathogens. The most aggressive fungal pathogen was *A. rolfsii* with a DS value of 4.8, and the less aggressive *S. sclerotiorum* with a DS value of 2.1. No DS values were recorded from fennel seedlings used as control not treated. The DS values recorded from leaves showed the same trend observed on the root, although they resulted in being lower than in those collected from roots (Table 2). The percentages of re-isolation of fungal soil-borne pathogens were significantly higher when the plants were not treated with both kinds CARA17 inoculations (100.00% for *A. rolfsii*; 80.00% for *V. dahliae*), while they were lower when the seedlings were treated with CARA17 by dipping inoculation (13.3% for all fungal pathogens except for *V. dahliae*), and were major with CARA17 inoculated by pouring into pots after transplanting (26.6% for *A. rolfsii*, and 80.00% for *V. dahliae*) (Table 2). 

### 2.5. Plant Growth Promotion by Streptomyces Strain In Vivo

Table 3 also reports the data related to the promotion of plant growth by the evaluation of fresh and dry biomasses (related to the entire plants). According to Shapiro–Wilk tests, the data from fresh and dry biomasses followed normal distributions (fresh biomass: W = 0.90, *p* < 0.01; dry biomass: W = 0.88; *p* < 0.01). The Levene tests showed that the homogeneity of variances, from the in vivo antagonism experiments (TEST1 and TEST2) carried out on fennel seedlings, were significant (fresh biomass: F = 3.74, *p* < 0.01; dry biomass: F = 3.75, *p* < 0.01). One-way ANOVA highlighted the inoculation by the CARA17 strain significantly promoting the growth of fennel seedlings also in the presence of fungal soil-borne pathogens.

The lowest biomasses produced (fresh, 38.13 g; dry, 4.15 g) were recorded from seedlings treated only with *A. rolfsii* with respect to other fungal soil-borne pathogens used alone without any antagonistic treatment (Figure 2). Indeed, the fresh/dried biomasses varied from 44.08/4.89 to 48.33/6.03 g, when the seedlings were treated with the other fungal soil-borne pathogens. In general, the biomasses resulted in being higher when the fennel seedlings were subjected to dipping in CARA17 inoculum rather than to being poured into pots after transplanting. In particular, the trials where the CARA17 strain was used as the dipping inoculation of seedlings inoculated with *A. rolfsii* as fungal soil-borne pathogen, allowed obtaining the highest biomasses (fresh, 93.11 g; dry, 11.69 g). The biomass values (fresh/dry) from other trials treated with the dipping of CARA17 inoculum and other fungal pathogens varied from 85.07/6.90 g (*F. oxysporum*) to 87.27/8.23 g (*S. sclerotiorum*).

The treatment of the CARA17 strain by dipping without fungal pathogens allowed the fennel seedlings to reach significantly higher biomass values (fresh/dry, 165.03/14.43 g) than during treatment after transplanting (fresh/dry, 122.86/12.74 g), and no antagonistic treatment (fresh/dry, 101.61/11.66 g) (Table 3).

## 3. Discussion

Since in agriculture, low environmental impact control means that phytopathogenic agents are increasingly in demand to reduce the negative effects of chemicals such as pesticide residues in plant products for human use and to preserve the natural ecosystems, the present work reports preliminary results obtained from biological assays carried out in in vivo and in vitro conditions. It is known that biological control by microorganisms such as *Streptomyces* spp. confirms that they are a promising tool for the management of various microbial diseases causing severe losses of agricultural yields. For this reason, the development of biological control (BCAs) and plant growth promoting agents are needed [19]. To date, a very large number of *Streptomyces* strains as antagonists have been used to promote plant growth and control soil-borne phytopathogens [20]. In the present work, a *Streptomyces* strain named CARA17 isolated from some healthy roots of grapevine affected by grapevine trunk diseases was subjected to identification and then used to ascertain its capacities to control five soil-borne phytopathogens, known to be ubiquitous and polyphagous. With cultural and molecular tools, the strain CARA17 was found to belong to the *Streptomyces albidoflavus* group, which was used to ascertain if it was able to control *A. rolfsii*, *F. oxysporum*, *P. ramiseptata*, *S. sclerotiourum* and *V. dahliae* as severe fungal phytopathogens. Antagonism assays carried out in dual cultures in in vitro conditions showed that the *Streptomyces* CARA17 strain was capable of inhibiting the mycelial growth of soil-borne pathogens used as targets with varying degrees of inhibition. As it was observed that the CARA17 strain could inhibit the mycelial growth after some days, in order to emphasize its antagonistic capability, it was placed in Petri dishes 14, 21 and 28 days before the placing of mycelia plugs of phytopathogens. Indeed, it was possible to assess that the CARA17 strain showed a high ability to control the fungal mycelium starting from 14 days after its placement in a Petri dish. It is probable that the CARA17 strain releases bioactive antimicrobic compounds in an artificial medium some days after its placement according to Kaur et al. [21], who found that *Streptomyces* spp. are able to produce a large number of metabolic compounds. In particular, these researchers assessed the antifungal activity of *Streptomyces* spp. against *Fusarium moniliforme* which is responsible for Fusarium wilt in tomato plants in both in vitro and in vivo experiments. Moreover, other investigations reported the effectiveness of *Streptomyces* strains for managing rice blast disease caused by the fungus *Magnaporthe oryzae* [22] in greenhouse conditions, and considering these actinobacteria excellent candidates as biocontrol agents. According to the latest research, our findings allowed us to observe that the CARA17 strain was able to inhibit *S. sclerotiorum* all three times (14, 21 and 28 days of exposition) while against the other fungal pathogens the antagonistic effectiveness was weakly reduced but still significant, and decreased weakly from 14 days to 28 days of exposition. In dual culture, only *A. rolfsii* was already poorly controlled at 14 days of exposition, and not inhibited at 21 and 28 days.

In in vivo conditions, the CARA17 strain was also demonstrated to be efficacious at controlling all fungal phytopathogens used in the present work, although the best effectiveness consisted of the control of *Scleotinia sclerotiorum* for both trials—the dual culture and the greenhouse experiment. Lower but significant biocontrol action acted on by the CARA17 strain was detected against *F. oxysporum*, *P. ramiseptata* and *V. dahliae*. Similar results have been discussed by Colombo et al. [15] when they used two *Streptomyces* strains to control *Fusarium graminearum* as an agent of FRR (Fusarium root rot), FFR (Fusarium foot rot) and FHB (Fusarium head blight) in greenhouse and open field conditions. While no protection was assessed against *A. rolfsii*, a considerable effect of growth promotion in fennel seedlings was observed. The biological control played by the CARA17 strain on fennel seedlings artificially inoculated with fungal soil-borne pathogens was more clear in terms of the protection of seedlings by dipping inoculation before transplanting than by pouring the inoculation into pots after transplanting. The CARA17 strain was probably more able to protect the root of fennel seedlings by dipping inoculation, because the antifungal compounds present in the inoculum solution were immediately adsorbed by roots enhancing the hyperparasitism mechanism and inducing systemic resistance [23,24].

Further, the greenhouse experiments determined significant effects of *Streptomyces albidoflavus* CARA17 strain on the growth parameters of the fennel seedlings, such as the hypogeal and epigeal portions and the fresh and dry weights of the biomasses. Therefore, the data obtained from the in vivo experiments suggested that the CARA17 strain might be used as a biological control agent (BCA) and plant growth promoting agent (PGPA) when used as a propagules inoculum containing spores or mycelium. It is known that *Streptomyces* spp. are included in the PGPR microbial community (Plant Growth Promoting Rhizobacteria), and they are actively or passively involved in plant growth promotion [25]. Therefore, in this work, by the fresh and dry biomass weights obtained, it was possible to assess the ability of the CARA17 strain: (a) to promote the fennel seedlings growth, because it may act as a biofertilizer; (b) to facilitate the tolerance to biotic (phytopathogens used in vivo conditions) and abiotic stresses (no organic and mineral fertilizers), because the disease severity indices collected from the fennel seedlings were significantly reduced due to the presence of the CARA17 strain.

Moreover, this study allowed us, for the first time, to associate *Plectosphaerella ramiseptata*, as a soil-borne pathogen, with fennel plants, and to ascertain its ability to cause significant damages to seedlings consisting of root browning, leaf yellowing and plant growth reduction. Similar symptoms and the disease severity caused by *P. ramiseptata* have also been reported in tomato, pepper, basil and parsley [26,27].

The preliminary results discussed here encourage further studies to assess whether the CARA17 strain is able to produce putative antifungal compounds, to extract secondary metabolites as putative resistance inducers, and to verify if biocontrol and growth promotion actions can be improved and increased by formulation with other PGPR microorganisms.

## 4. Materials and Methods

### 4.1. Isolation and Identification of Actinomycetes Strains

During a survey carried out from 2015 to 2019 on the root diseases of grapevine plants affected by decline and apoplexy, caused by fungal agents of grapevine root (Black foot) such as *Dactylonectria* spp., *Ilyonectria* spp. and trunk diseases (GTD) such as *Phaeomoniella chlamydospora* and *Phaeoacremonium* spp. [28], other unknown microorganisms were isolated including not-sporulated fungi, bacteria and *Actinomycetes*. Among the latter microbial agents, a consistent number (37 isolates, corresponding to 1.6% of microorganisms isolated) of presumptive *Streptomyces* strains were observed and recorded. All *Actinomycetes* cultures were subjected to purification techniques by spreading over Petri dishes of agar and water (AW). After an overnight incubation at 28 ± 3 °C, single germinating spores or small pieces of hyphae were transferred to Petri dishes with fresh potato-dextrose-agar (PDA; 39 g/L, Oxoid) for molecular identification. For this purpose, a representative strain of *Actinomycetes* showing a putative antimicrobial activity, called CARA17, was used for molecular characterization. Genomic DNA of the CARA17 strain was extracted from a 15-day-old culture growing on PDA at 28 ± 3 °C in the dark, according to Carlucci et al. [29].

For preliminary molecular identifications, the primer pairs used were 16SAct1F (5′ CGC GGC CTA TCA GCT TGT TG 3′) and 16SAct1R (5′ CCG TAC TCC CCA GGC GGG G 3′) of 16S rDNA for the amplification of the 16S ribosomal RNA (R RNA) region [30]. The amplification was made according to the following PCR protocol: 1× PCR buffer, 2.5 mM MgCl2, 200 µM of each nucleotide, 2.5 pmol of each primer, 0.25 U Taq polymerase, 0.5 µL DMSO, and a 30–50 ng DNA template taken to a total volume of 25 µL. The Taq polymerase, nucleotides and buffers were supplied by Eurofins Genomics (Milan, Italy). The amplification conditions were: initial denaturation for 5 min at 94 °C, followed by 35 cycles of denaturation for 1 min at 95 °C, annealing for 30 s at 58 °C, elongation for 1 min at 72 °C and the final extension step for 10 min at 72 °C. Five microliters of amplicon were analyzed by electrophoresis at 100 V for 30 min in 1.5 % (*w/v*) agarose gels in 1× TAE buffer (40 mM Tris, 40 mM acetate, 2 mM EDTA, pH 8.0). The gels were stained with ethidium bromide and were visualized in a Gel Doc EZ System under UV light (Biorad, Hercules, CA, USA). The PCR products were purified before DNA sequencing, using Nucleo Spin Extract II purification kits (Macherey-Nagel, Germany), according to the manufacturer’s instructions. Both strands of the PCR products were sequenced by Eurofins Genomics (Ebersberg, Germany). The nucleotide sequences obtained were manually edited using BioEdit v.7.0.9 (http://www.mbio.ncsu.edu/ BioEdit accessed on 8 Febrary 2021). The consensus sequence was compared with those available in the GenBank database, using the Basic Local Alignment Search Tool (BLAST, http://www.ncbi.nlm.nih.gov/ accessed on 24 March 2021) to confirm the preliminary morphological identification and to ascertain the sequence similarity searches.

### 4.2. Fungal Soil-Borne Phytopathogens

To assess the antagonistic activity of the *Streptomyces* strain CARA17 in vitro and in vivo conditions, fungal soil-borne pathogens such as *Athelia rolfsii*, *Fusarium oxysporum*, *Sclerotinia sclerotiorum* and *Verticillium dahliae* strains were used, which were all isolated from fennel plants during previous surveys carried out in the northern Apulia region (2017–2020). The taxonomic identity of all fungal species here used was assessed earlier with molecular tools and DNA extraction according to Carlucci et al. [29] (data not shown).

From the root and collar of fennel plants, a conspicuous amount of isolates morphologically attributed to *Plectosphaerella* genus were collected; a representative strain was included in the in vitro and in vivo trials. For this purpose, the collection of *Plectosphaerella* isolates was subjected to DNA extraction, as mentioned above, and to molecular screening by MSP-PCR using the M13 minisatellite primer (5′-GAGGGTGGCGGTTCT-3′) [31]. MSP-PCR profiles were generated according to Santos and Phillips [32]. The DNA banding patterns were analyzed using the Bionumerics v.5.1 software (Applied Maths, A Biomerieux company, Sint-Martens-Latem, Belgium), with calculations of Pearson’s correlation coefficients and the unweighted pair group method with arithmetic means. The reproducibility levels were calculated by comparing the banding profiles obtained for the M13 primer. For this purpose, 10% of the strains were chosen from any cluster at random, and their profiles were analyzed again. The MSP dendrogram generated one clade, from which one isolate was chosen as a representative and was molecularly characterized according to Carlucci et al. [33] (data not shown).

All fungal strains used here are maintained in the laboratory of the Plant Pathology and Diagnosis of Department of Sciences Agriculture, Food, Natural resources and Engineering (DAFNE) at the University of Foggia, Italy.

### 4.3. Inhibitory Activity against Fungal Pathogens in Dual Cultures

An agar-mycelium disc (5 mm diameter) of CARA17, taken from the edge of a 21-day-old colony grown on PDA, was put in a PDA Petri dish at 15 mm from the center and was left to grow at 25 ± 3 °C in darkness. After 14, 21 and 28 days incubation, an agar-mycelium disc (5 mm diameter) from each fungal pathogen was put at 15 mm from the center in front of the agar disc with the CARA17 strain. Five replicates were performed for each fungal strain, and only the plates inoculated with the pathogen and the sterile agar disk were used as a control. The dual cultures were kept at 21 ± 3 °C for 15 days in darkness. The inhibitory activity (IA) was calculated as the percentage of mycelium growth inhibition compared to the control by the formula [(R1-R2)/R1] × 100, where R1 was the radius measurement from the center of the colony of fungal pathogen towards the edge of the control Petri plate (without CARA17), and R2 was the radius from the center towards the edge of the fungal colony in the direction of the antagonist CARA17, respectively, according to Kunova et al. [34].

The percentage data of inhibition activity were arcsine root-square transformed in Excel 2007 by the formula DEGREES(ASIN(SQRT(X))), where X is the percentage value. One-way ANOVA analysis was performed using Statistica, version 6 (StatSoft, Hamburg, Germany) to assess the significant differences of inhibition activity values. Fisher’s test was used as a post-hoc test (*p* = 0.01).

### 4.4. Assessment of Toxicity/Pathogenicity of CARA17 Strain on Cucumis sativus L. Cotyledons In Vitro

The *Streptomyces* strain CARA17 was assayed for its putative ability to cause damage to horticultural crops by the inoculation of a suspension of its propagules (solution at 0.2% of Tween 20 in sterile water) on young cucumber cotyledons. The cotyledons were taken from seeds germinated on peat loam sterilized at 121 °C for 30 min three times at intervals of 24 h. They were gently disinfected by immersion in 70 % ethanol for 5 min, rinsed in sterile distilled water and dried on sterile paper. Four disinfected cotyledons were gently placed in Petri dishes containing water-agar (0.3%) and were inoculated with a drop of 20 µL of *Streptomyces* strain CARA17 propagules suspension at 1 × 10^6^ cfu/mL. As a control assay, the cotyledons were inoculated with sterile Tween 20 solution (0.2%). This assay was replicated six times and kept at 21 ± 3 °C in darkness, and the cotyledons were inspected after 3, 6, 9 and 12 days to ascertain putative toxicity and/or pathogenicity symptoms.

### 4.5. Assessment of Antagonistic Effectiveness of CARA17 Strain on Foeniculum vulgare L. Seedlings against Fungal soil-borne pathogens In Vivo

The inoculum solution with the CARA17 strain propagules was prepared by collecting spores and small fragments of mycelium scraped from surface of 21-day-old *Streptomyces* colonies grown on PDA medium at 28 ± 3 °C in darkness until reaching a concentration of 1 × 10^7^ cfu/mL in sterile Tween 20 solution (0.2%).

*Inoculation preparation of fungal soil-borne pathogens.* The inoculum solution with each fungal soil-borne pathogen was prepared as described above for the CARA17 strain, scraping from the surface of 21-day-old colonies grown on PDA medium at 21 ± 3 °C in darkness until reaching a concentration of 1 × 10^7^ cfu/mL in sterile Tween 20 solution (0.2%).

The experimental design was performed as two independent batches at the end of August and consisted of two different inoculation kinds, where the horticultural host target was represented by 30-day-old seedlings of *Foeniculum vulgare* var. DONATELLO F1 (HM.CLAUSE Vegetable Seeds).

The first experiment (TEST1) consisted of preliminarily dipping the fennel seedlings in the inoculum solution of the CARA17 strain for 30 min, before transplanting them in a pot containing 1.5 kg of soil and peat (3:1), (sterilized early twice at 121 °C for 30 min and kept for 20 days in a controlled chamber at 25 ± 3 °C, 70% relative humidity, and under natural light), and wetting them with 500 mL of irrigation water. Subsequently, 50 mL of inoculum solution of each fungal soil-borne pathogen was poured into the soil of each pot around the collar of the fennel seedlings.

The second experiment (TEST2) consisted of inoculation with 50 mL of inoculum solution of each fungal soil-borne pathogen after seedlings transplantation into wet soil with 500 mL of irrigation water. After 48 h, 50 mL of CARA17 inoculum solution was poured into the soil around the collar of the fennel seedlings.

As control trials, for both experiments (TEST1 and TEST2), pots containing fennel seedlings treated with the CARA17 strain, treated with each fungal pathogen, and not treated with either the CARA17 strain or the fungal soil-borne pathogens, were prepared. Each trial was replicated fifteen times. The pots with fennel seedlings were placed in a greenhouse with temperature and humidity not conditioned. During the growth of the seedlings, no fertilizers, pesticides or fungicides were used. They were only subjected to irrigation practice when necessary, using the same water volumes for each pot. After 100 days, the fennel plants were gently removed from the pots, the roots and collars were carefully washed, and the presence/absence of browning symptoms observed on the root and collar were evaluated and described using an empiric scale from 0 to 5, where 0 = no symptoms observed; 1 = 1–20%; 2 = 21–40%; 3 = 41–60%; 4 = 61–80%; and 5 = 81–100%. The disease severities (DS) on the roots and collar were determined according to the following formula:

(1) $$\mathrm{DS}=\frac{\sum \left(Number\;of\;observ\times values\;of\;scores\right)}{Total\;number\;of\;cases}.$$

All fungi underwent re-isolation from the root, collar and stem of the inoculated plants to fulfil Koch’s postulates.

### 4.6. Plant Growth Promotion by Streptomyces Strain In Vivo

To assess putative plant growth promotion by the CARA17 strain on fennel plants, during both the experiments described above (TEST1 and TEST2), all plants were cut at the basis of the collar, and all tissues of epigeal (collar, stem and leaves) and hypogeal portions (root), after a careful washing, were separately weighed and put into a stove at 105 °C until reaching a constant dry weight. For all trials, the average weight of 15 fennel plants (epigeal and hypogeal portions) were calculated. To determine whether these data followed normal distributions, the Shapiro–Wilk test (W test) was used. The homogeneity of variance of the datasets was assessed on the basis of fresh and dried weights of fennel seedlings using the Levene test. One-way ANOVA was performed using Statistica v. 6 (StatSoft, Hamburg, Germany) to determine the significant differences in fresh and dried weights recorded during both kinds of antagonist inoculation (by dipping and by pouring after transplanting). Fisher’s test was used for the comparison of the treatment means, at *p* < 0.01.

## Figures and Tables

**Table 1:** Inhibition activity percentage (IA) by CARA17 strain against mycelial growth of soil-borne pathogens in vitro (dual cultures).

	Inibition Activity (IA) %					
Fungal Soilborne Isolates	14 Days ^a^		21 Days		28 Days	
	Mean	Min–Max ^c^	Mean	Min–Max	Mean	Min–Max
*Athelia rolfsii*	15.08 D ^b^	14.92–15.25	4.31 D	4.12–4.50	0.00 D	0.00–0.00
*Verticillium dahliae*	87.42 C	86.31–86.54	77.15 BC	76.96–77.34	71.30 C	71.28–71.32
*Plectosphaerella ramiseptata*	96.35 B	96.33–96.74	84.82 B	84.58–85.07	82.73 B	82.27–83.19
*Fusarium oxysporum*	88.26 D	88.63–87.89	84.91 B	82.32–87.50	84.71 B	80.82–88.60
*Sclerotinia sclerotiorum*	100.00 A	100.00–100.00	100.00 A	100.00–100.00	100.00 A	100.00–100.00

^a^ Exposition of fungal strains vs. CARA17 grown on medium at different colony ages; ^b^ Data followed by different capital letters within the column are significantly different (Fisher’s tests; *p* < 0.01); ^c^ Minimum and maximum values detected (five observations).

**Table 2:** Effectiveness of CARA17 strain as antagonist for controlling disease severity (DS) against fungal soil-borne pathogens on fennel seedlings.

			Description Symptoms Recodered				
	Treatment with CARA17 Strain	Fungal Pathogen	Root	DS	Leaves	DS	Re-Isolation from Root (%)
**TEST 1**	Dipping	*Athelia rolfsii*	No disease symptoms	0.0	No disease symptoms	0.0	13.3
	Dipping	*Fusarium oxysporum*	Light root browning	0.9	Light growth reduction and yellowing	0.6	13.3
	Dipping	*Plectosphaerella ramiseptata*	Light root growth reduction	0.6	Light apical yellowing	0.4	13.3
	Dipping	*Sclerotinia sclerotiorum*	No disease symptoms	0.0	No disease symptoms	0.0	13.3
	Dipping	*Verticillium dahliae*	Light root growth reduction	0.8	Light growth reduction	0.7	26.6
	Dipping	No fungal pathogen	No disease symptoms	0.0	No disease symptoms	0.0	-
**TEST 2**	After transplanting	*Athelia rolfsii*	No disease symptoms	0.0	No disease symptoms	0.0	26.6
	After transplanting	*Fusarium oxysporum*	Root growth reduction	1.9	Growth reduction and yellowing	1.2	40.0
	After transplanting	*Plectosphaerella ramiseptata*	Root browning	1.9	Growth reduction and yellowing	1.5	86.6
	After transplanting	*Sclerotinia sclerotiorum*	Light root growth reduction	0.6	No disease symptoms	0.0	33.3
	After transplanting	*Verticillium dahliae*	Root growth reduction	1.8	Growth reduction	1.8	80.0
	After transplanting	No fungal pathogen	No disease symptoms	0.0	No disease symptoms	0.0	-
	No treatment	*Athelia rolfsii*	Root browning and rot	4.8	Growth reduction and yellowing	2.7	100.0
	No treatment	*Fusarium oxysporum*	Root browning	2.7	Growth reduction and yellowing	1.8	86.6
	No treatment	*Plectosphaerella ramiseptata*	Root browning	2.9	Growth reduction and yellowing	1.7	93.3
	No treatment	*Sclerotinia sclerotiorum*	Root browning and rot	2.1	Growth reduction	1.4	93.3
	No treatment	*Verticillium dahliae*	Root growth reduction	2.3	Growth reduction	1.5	80.0
	Control	No fungal pathogen	No disease symptoms	0.0	No disease symptoms	0.0	-

**Table 3:** Effectiveness of CARA17 strain on promotion of plant growth by biomass production of fennel seedlings.

			Growth Promotion by Biomass * (gr)			
			Fresh		Dry	
	Treatment with CARA17 Strain	Fungal Pathogen	Mean **	SD ^§^	Mean	SD
**TEST 1**	Dipping	*A. rolfsii*	93.11 E ^†^	5.13	11.69 D	2.64
	Dipping	*F. oxysporum*	85.07 D	3.97	6.90 B	0.83
	Dipping	*P. ramiseptata*	85.57 D	4.14	7.15 BC	1.71
	Dipping	*S. sclerotiorum*	87.27 D	7.04	8.23 C	0.92
	Dipping	*V. dahliae*	85.15 D	3.33	6.86 B	0.99
	Dipping	No fungal pathogen	165.03 H	15.60	14.43 E	2.47
**TEST 2**	After transplanting	*A. rolfsii*	69.95 C	10.02	8.81 C	2.13
	After transplanting	*F. oxysporum*	60.05 C	2.37	6.25 B	0.57
	After transplanting	*P. ramiseptata*	45.20 A	7.00	4.93 AB	0.61
	After transplanting	*S. sclerotiorum*	87.27 D	5.29	7.35 BC	0.99
	After transplanting	*V. dahliae*	69.97 C	11.14	6.34 B	2.16
	After transplanting	No fungal pathogen	122.86 G	15.76	12.74 DE	1.74
	No treatment	*A. rolfsii*	38.13 A	5.69	4.15 A	0.34
	No treatment	*F. oxysporum*	47.69 AB	3.69	5.09 AB	0.24
	No treatment	*P. ramiseptata*	48.33 AB	4.56	6.03 B	0.61
	No treatment	*S. sclerotiorum*	44.08 A	4.68	4.89 AB	0.70
	No treatment	*V. dahliae*	45.51 A	6.20	5.00 AB	0.29
	Control	No fungal pathogen	101.61 F	7.00	11.66 D	1.61

* Biomass includes epigeal and hypogeal plant tissues; ** mean values of 15 replicates; ^§^ Standard Deviation; ^†^ Data followed by different capital letters within the column are significantly different (Fisher’s tests; *p* < 0.01).

## Data Availability

Not applicable.
